# Solitary Palmar Keratoacanthoma: Case Report

**DOI:** 10.7759/cureus.2331

**Published:** 2018-03-15

**Authors:** Raghavendra L Girijala, Young Kwak, David Wright, Leonard H Goldberg

**Affiliations:** 1 College of Medicine, Texas A&M; 2 Dermatology, Derm Surgery Associates, PA

**Keywords:** palmar, keratoacanthoma, non-follicular, trauma, mohs micrographic surgery

## Abstract

Keratoacanthoma (KA) is a squamous neoplasm exhibiting a triphasic growth pattern involving rapid growth, stabilization, and eventual spontaneous resolution. Historically, keratoacanthomas were thought to originate on hair-bearing skin or sun-exposed surfaces. However, recent reports demonstrate that they can occur on the mucous membranes, subungual regions, and palms and soles. We report a 74-year-old man who developed a KA on the left palmar surface after minor trauma, for which he underwent Mohs’ micrographic surgery. A literature review for the terms: keratoacanthoma, palm, palmar, volar, plantar, and sole resulted in only four reported cases of solitary or giant KA of the palms and soles; excluding our patient, all of the cases occurred on the plantar foot. A number of reports describe palmar KA in the context of multiple lesions occurring simultaneously. However, to our knowledge, our patient represents the first reported case of a solitary palmar KA in the literature. The features of follicular and non-follicular keratoacanthomas (KAs) and their association with trauma are discussed.

## Introduction

Keratoacanthoma (KA) is a common squamous neoplasm of uncertain malignancy characterized by rapid growth over the course of two to four weeks and subsequent spontaneous resolution [1]. While predisposing genetic factors have yet to be implicated in the pathogenesis of solitary KA, numerous risk factors have been observed, including but not limited to immunosuppression, ultraviolet radiation, chemical irritants, foreign bodies, medications, and trauma [1]. We report a case of a 74-year-old man who presented with KA of the left palm after minor trauma in the same area. Given rare reports of malignant degeneration into squamous cell carcinoma and the possibility of developing a giant KA destroying local tissue, he underwent definitive surgical management [1]. To our knowledge, only four other cases of solitary or giant KA of the palms and soles have been reported, all of which occurred on the plantar foot [2-4]. In contrast, cases of palmar KA have been reported only in the context of multiple KAs arising simultaneously [5-6].

## Case presentation

A 74-year-old man presented with a four-week history of a painless nodule in the center of his left palm that had been increasing in size. He had no chronic medical conditions and was not taking any medications. His past dermatologic history was significant for numerous squamous cell carcinomas, basal cell carcinomas, and keratoacanthomas that occurred individually over the course of multiple years. None of the prior lesions were located on the palmar or plantar surfaces, and all lesions had been definitively treated with Mohs’ micrographic surgery with no report of recurrence.

Upon questioning, it was found that the current lesion was initially noticed several days after having removed a splinter in the involved location of the left palm. No other history of trauma was reported. The patient also denied any systemic symptoms, including fevers, chills, weight loss, fatigue, cough, shortness of breath, dysphagia, hematuria, and changes in bowel habits. There was no history of immunosuppression, ultraviolet light therapy, smoking, or illicit drug use.

**Figure 1:**
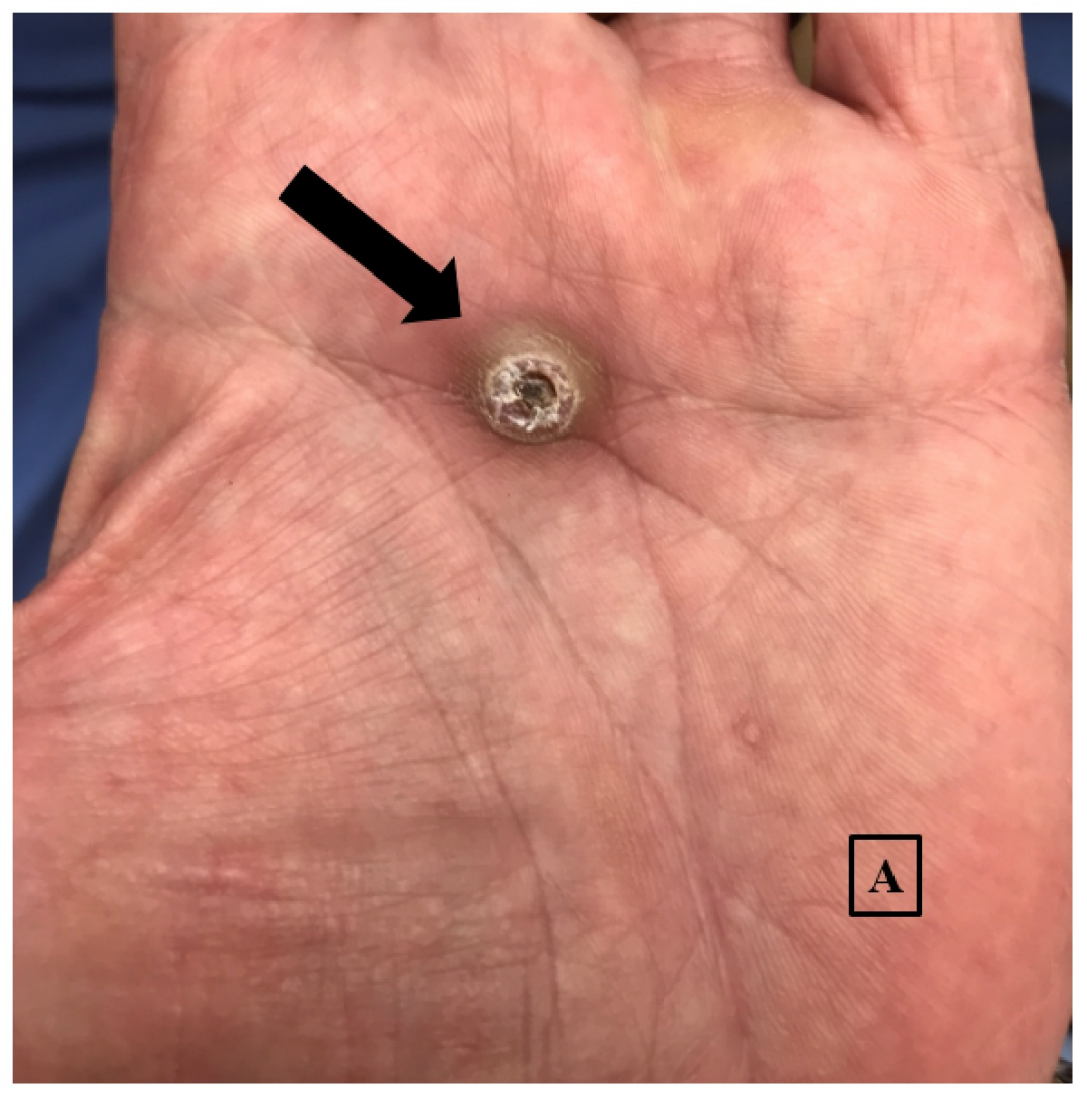
Palmar keratoacanthoma Solitary, firm 1.5 x 1.2 cm crateriform nodule in the center of the left palm

**Figure 2:**
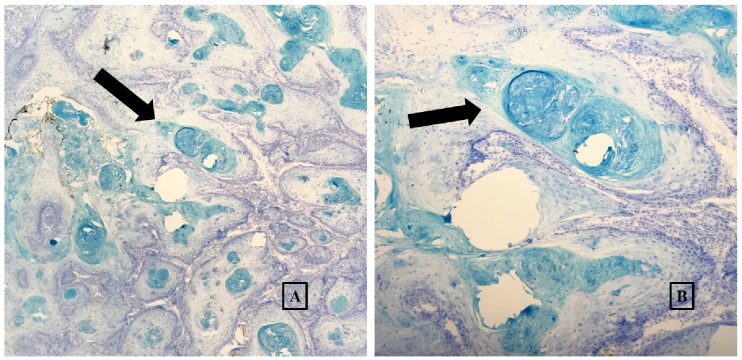
Pathology features of keratoacanthoma A well-defined nodular invasive squamous neoplasm with a cup-shaped invagination on the periphery. Well-differentiated neoplastic glassy squamous epithelioid cells arranged in a haphazard pattern surrounding multiple islands of keratin, consistent with keratoacanthoma, depicted in 40x (A) and 100x (B) magnification views

Cutaneous examination demonstrated a solitary 1.5 x 1.2 centimeter firm, round, and dome-shaped nodule with a hyperkeratotic core within a crateriform ulceration on the central aspect of the left palm (Figure 1). The remainder of the examination was unremarkable for similar or other concerning lesions. Histological assessment demonstrated a well-defined nodular invasive squamous neoplasm with a cup-shaped invagination on the periphery. Neoplastic glassy squamous epithelioid cells were overall well-differentiated and arranged in a haphazard pattern surrounding multiple islands of keratin. Perineural involvement, lymphovascular invasion, and tumor necrosis were not observed on the sections examined. The overall features were consistent with a keratoacanthoma (Figure 2). No foreign body was identified on pathology. Histology margins were cleared with Mohs’ micrographic surgery, and the patient is being followed regularly for recurrence or emergence of other cutaneous lesions.

## Discussion

Solitary KAs most often present on sun-damaged skin and can present anywhere on the body but generally spare the palms, soles, and mucous membranes [1]. Historically, these neoplasms were thought to derive from the hair-follicles given that their lifecycle mimicked the anagen (growth), telogen (rest), and involution (catagen) phases of the follicular cycle [7]. This theory was further supported by KAs demonstrating markers consistent with follicular isthmic and infundibular tissue [1].

However, a small but growing number of reports have documented solitary KAs on the conjunctiva, vulva, subungual region, soles, and with our patient, the palm [1]. As a result, the cell of origin for these tumors has been reconsidered; currently, KA is classified as follicular or non-follicular [7]. Notably, involution has not yet been observed in a non-follicular KA developing in the mucous membranes, subungual area, or palms and soles [7]. Therefore, it is unclear if the lifecycle of non-follicular KA is simply extended or if it demonstrates cell immortality, as with most cancers.

The differences between follicular and non-follicular KAs extend beyond lifecycle. Unlike follicular KA, non-follicular KA is not necessarily crateriform and can be painful, especially when presenting in the subungual region [7]. On histopathology, non-follicular KA tends to have fewer inflammatory cells but also presents with more dyskeratosis. Finally, it does not demonstrate fibrosis at its base, which is consistent with its lack of involution when compared to follicular KA [7].

Including our patient, only five cases of solitary or giant KA of the palms and soles are reported in the literature (Table 1) [2-4]. Three males and two females are reported. Ages ranged from 38 to 74 years, with 80% of cases occurring in patients over 60 years of age. The median age was 65 years. The majority of cases occurred on the plantar surface (80%) and 40% of cases were classified as giant KA, measuring greater than 20 millimeters.

**Table 1:** Characteristics of five patients with solitary or giant keratoacanthoma of the palms and soles. cm: centimeter; CR: current report; F: female; M: male; Mohs: Mohs micrographic surgery; N: no; Y: yes.

Case	Age, Gender	Location	Size (cm)	Treatment	Trauma	Reference
1	61, F	Sole	1.3 x 1.2	Wide excision	N	[4]
2	38, F	Sole	2.0 x 2.0	Wide excision	N	[2]
3	65, M	Sole	2.4 x 2.8	Wide excision	Y	[3]
4	70, M	Sole	2.2 x 3.0	Wide excision	Y	[3]
5	74, M	Palm	1.5 x 1.2	Mohs	Y	CR

Trauma related to KA is classified as iatrogenic or non-iatrogenic. The former can include procedures, such as surgery, microdermabrasion, cryotherapy, photodynamic therapy, and chemical peels; the latter includes environmental trauma and tattoos [1]. A study by Ghadially et al. followed 238 patients and ascertained that 25 patients (10.5%) developed KA in association with a cutaneous injury secondary to trauma or skin pathology causing epithelial disruption; lesions developed from one week to one year after trauma [8]. Sixty percent of cases in Table 1 documented associated trauma prior to the onset of a non-follicular KA, implying a potential role of trauma in their development. Therefore, a potential explanation for the higher number of plantar non-follicular KA cases reported is that activities of daily living are more likely to cause traumatic injury to the sole of the foot than to the palm of the hand.

The management of solitary follicular KA is centered on surgical treatment for a number of reasons. While it is generally understood that KAs eventually regress, the final size of the mass and timeframe of growth are unpredictable; therefore, potentially disfiguring lesions can be produced in the interim [1]. It may also be difficult to distinguish KAs from invasive squamous cell carcinomas because both can present as firm red nodules with potential invasion and keratin scale; some authors even refer to KAs as self-healing squamous cell carcinoma [1]. The challenge in differentiating the two clinical entities is present even with the use of dermoscopy and histopathological examination [1]. Furthermore, KA metastasis has been reported, indicating that these tumors may have more malignant potential than previously understood [9].

Multiple KAs involving the palms have been effectively treated with systemic agents, such as acitretin and methotrexate [5-6]. In the case of a solitary KA of the palms and soles, surgical management is the gold standard [1]. A study by Larson et al. followed 42 biopsy-proven KAs treated with Mohs micrographic surgery with the fresh-tissue technique from six months to two years; only one case of recurrence (2.4%) was noted [10]. In addition to the well-documented benefits of tissue preservation in Mohs’ micrographic surgery, this therapeutic method may help limit the risk of further iatrogenically-induced KAs. Recurrence rates from other techniques, including wide local excision, range from 1 to 8% [1].

## Conclusions

Solitary KA is a neoplasm most commonly affecting hair-bearing skin. Including our patient, only five reports of solitary or giant KA affecting the palms and soles are reported in the literature. A potential association between trauma and non-follicular KA may exist; further reports are needed to define a relationship between epidermal injury and a particular subtype of KA. Given the potential for misdiagnosis or metastasis in rare cases, these lesions should be managed definitively.
